# Array-Comparative Genomic Hybridization Reveals Loss of *SOCS6* Is Associated with Poor Prognosis in Primary Lung Squamous Cell Carcinoma

**DOI:** 10.1371/journal.pone.0030398

**Published:** 2012-02-17

**Authors:** Krishna B. Sriram, Jill E. Larsen, Santiyagu M. Savarimuthu Francis, Casey M. Wright, Belinda E. Clarke, Edwina E. Duhig, Kevin M. Brown, Nicholas K. Hayward, Ian A. Yang, Rayleen V. Bowman, Kwun M. Fong

**Affiliations:** 1 School of Medicine, The University of Queensland, Brisbane, Queensland, Australia; 2 Department of Thoracic Medicine, The Prince Charles Hospital, Brisbane, Queensland, Australia; 3 Hamon Center for Therapeutic Oncology Research, University of Texas Southwestern Medical Centre, Dallas, Texas, United States of America; 4 Department of Anatomical Pathology, The Prince Charles Hospital, Brisbane, Queensland, Australia; 5 Integrative Cancer Genomics Division, Translational Genomics Research Institute (TGen), Phoenix, Arizona, United States of America; 6 Oncogenomics Laboratory, Queensland Institute of Medical Research, Brisbane, Queensland, Australia; University of Kansas Medical Center, United States of America

## Abstract

**Background:**

Primary tumor recurrence commonly occurs after surgical resection of lung squamous cell carcinoma (SCC). Little is known about the genes driving SCC recurrence.

**Methods:**

We used array comparative genomic hybridization (aCGH) to identify genes affected by copy number alterations that may be involved in SCC recurrence. Training and test sets of resected primary lung SCC were assembled. aCGH was used to determine genomic copy number in a training set of 62 primary lung SCCs (28 with recurrence and 34 with no evidence of recurrence) and the altered copy number of candidate genes was confirmed by quantitative PCR (qPCR). An independent test set of 72 primary lung SCCs (20 with recurrence and 52 with no evidence of recurrence) was used for biological validation. mRNA expression of candidate genes was studied using qRT-PCR. Candidate gene promoter methylation was evaluated using methylation microarrays and Sequenom EpiTYPER analysis.

**Results:**

18q22.3 loss was identified by aCGH as being significantly associated with recurrence (*p* = 0.038). Seven genes within 18q22.3 had aCGH copy number loss associated with recurrence but only *SOCS6* copy number was both technically replicated by qPCR and biologically validated in the test set. *SOCS6* copy number loss correlated with reduced mRNA expression in the study samples and in the samples with copy number loss, there was a trend for increased methylation, albeit non-significant. Overall survival was significantly poorer in patients with *SOCS6* loss compared to patients without *SOCS6* loss in both the training (30 vs. 43 months, *p* = 0.023) and test set (27 vs. 43 months, *p* = 0.010).

**Conclusion:**

Reduced copy number and mRNA expression of *SOCS6* are associated with disease recurrence in primary lung SCC and may be useful prognostic biomarkers.

## Introduction

Lung cancer is the leading cause of cancer-related mortality worldwide, accounting for greater than one million deaths annually [1]. Non-small cell lung cancer (NSCLC) accounts for 80% of all lung cancer diagnoses. Conventionally NSCLC has been divided into three subtypes: adenocarcinoma (AC), squamous cell carcinoma (SCC) and large cell carcinoma (LC), with AC and SCC accounting for 85% of NSCLC cases [2]. The treatments for NSCLC have been generic and largely ineffective resulting in a five-year survival of 15% [3]. Early diagnosis followed by surgical resection remains the most effective treatment strategy [4]. However, even in stage I patients undergoing surgical resection, recurrence of the primary tumor occurs in 30–35% of cases [2]. Molecular alterations are likely to be involved in driving disease recurrence, but the specific genes involved remain to be elucidated.

DNA copy number alterations are ubiquitous to almost all human malignancies [5]. The identification of tumor-specific DNA copy number alterations can assist in the discovery of oncogenes or tumor suppressor genes which are typically located within genomic regions of amplifications or loss respectively [5]. Array-comparative genomic hybridization (aCGH) has been used to investigate copy number alterations in several malignancies, including lung SCC [6], [7], [8], [9], [10], [11], [12], [13], [14], [15], [16]. Karyotyping and conventional CGH characterized lung SCCs, as having frequent copy number gains in 1p, 3q, 5p, 7q and 8q and copy number loss in 3p, 5q, 8p, 9p, and 14q [13], [17], [18], [19], [20], [21], [22]. High-resolution copy number characterization of these regions resulted in the discovery of the driver oncogenes *SOX2* [14] and *FGFR1* [23]. To our knowledge, the few studies that have specifically evaluated genomic differences unique to lung SCC recurrence and/or metastases have used low-resolution platforms on relatively small sample sizes [18], [24]. Consequently, while these studies have identified genomic regions with copy number alterations associated with recurrence and/or metastases, they have not been able to identify the driving gene/s associated with recurrence.

In this study we analyzed lung SCC tumors using a whole-genome aCGH microarray platform to identify genomic copy number alterations specific to tumors, which developed early recurrence of primary tumor post-resection. To identify recurrence specific genes within the candidate genomic regions, we used an independent method of copy number determination (quantitative PCR) and confirmed the findings in an independent set of SCC tumors. Finally, to assess whether candidate gene/s copy number alterations have prognostic value, we analyzed the survival data of training and test set subjects.

## Materials and Methods

### Subjects and Tumor Samples

The training set consisted of sixty-two tumor samples, which were collected from patients with histologically proven primary lung SCC. The tumor samples were obtained from patients who underwent curative-intent surgical resection at The Prince Charles Hospital between 1990 and 2004. Formalin fixed paraffin embedded tissue samples of normal lung and tumor tissue, adjacent to the frozen tumor sample, was used for hematoxylin and eosin examination and only those tumor samples that contained at least 50% tumor cells and all surgical bronchial margins were free of disease were used as training set samples and underwent aCGH experiments. The subjects were fitted to one of our two disease recurrence outcome criteria: non-recurrence, clinically disease-free for at least 36 months following surgery; or recurrent disease, unambiguous clinical, imaging, or histopathologic evidence of local or distant recurrence of the original primary lung cancer in a local or distant metastatic site occurring between 3 and 18 months post-resection. The threshold of 36 months for non-recurrence cases was selected since most patients develop disease recurrence within this period of time and to allow for comparison with other similarly designed studies. In addition, an independent test set, consisting of seventy-two tumor samples that were collected and stored in The Prince Charles Hospital Lung Tissue Bank were utilized for validation purposes. Inclusion and exclusion criteria for the test set were identical to that used for the training set.

### Ethics Statement

Ethics approval was granted from TPCH Human Research Ethics Committee (HREC/09/QPCH/17) and The University of Queensland (Project number: 2009000727) and all subjects provided written informed consent prior to inclusion in the study.

### Nucleic Acid Extraction

Tumor and paired normal lung tissue collected from surgical resection specimens, as previously described [25], were snap frozen and processed for genomic DNA and total RNA extraction. A total of 300–500 mg of frozen tissue was used for genomic DNA extraction using a modified salt-precipitation method [26]. High molecular weight genomic DNA was purified using a Blood and Cell Culture mini kit (Qiagen, Hilden, Germany) following the manufacturer's instructions. Purified genomic DNA was quantitated with a NanoDrop spectrophotometry system (NanoDrop Technologies, Wilmington, DE, USA) using 2 µL of DNA. Total RNA was extracted as described previously [25].

### Array Comparative Genomic Hybridization

aCGH experiments were performed on Human Genome Microarray 44B (Agilent Technologies Inc.) microarrays, a high-resolution 60-mer oligonucleotide-based microarray that contains 42,920 probes sourced from the NCBI human genome reference sequence. These probes represent 24,983 genes. Microarray images were analyzed using Feature Extraction Software, version 8.0 (Agilent Technologies, Inc.) and assessed for relative data quality in CGH Analytics, version 3.4.27 (Agilent Technologies, Inc.). For each aCGH microarray, a tumor sample (test) was compared with a commercially available Female Genomic DNA (Promega, Madison, USA). A triangular smoothing algorithm with a moving average window of 2 Mb was applied to log ratio aCGH data. aCGH microarray experiments were designed in compliance with the MIAME guidelines (http://www.mged.org/Workgroups/MIAME/miame.html). The aCGH raw data (unchanged), metadata and clinical information of the subjects in this study have been deposited in the NCBI Gene Expression Omnibus (GEO) public repository (http://www.ncbi.nlm.nih.gov/geo) and can be accessed through the accession number GSE32058. Normalized aCGH data was analyzed using Genomic Identification of Significant Target in Cancer (GISTIC), a bioinformatics method that identifies genomic regions most likely to contain oncogenes and tumor suppressor genes [27]. Segmented regions were estimated with Circular Binary Segmentation (CBS) using the R package “DNACopy” (http://www.r-project.org/). Copy number variation data from the Human Genome Build 35 (hg17) was obtained from the Database of Genomic Variants (DGV, http://projects.tcag.ca/variation). GISTIC scores for locus (*G* score) were obtained as the product of frequency and mean amplitude of amplifications or deletions. Only amplifications exceeding a log_2_ copy number ratio of 0.848 for amplifications or below 0.737 for deletions were included, accounting for 2.8 copies per cell and 1.6 copies per cell in samples respectively. *G* scores were compared against a null model to determine a false discovery rate (*q* value) and peaks with *q* values below 0.05 were considered.

### Real-time Quantitative PCR and Quantitative Reverse Transcription PCR

To validate copy number alterations detected by aCGH, real-time quantitative PCR (qPCR) assays were used. Pre-designed QuantiTect primers (Qiagen) were used to measure candidate gene copy number by qPCR, details of which are provided in Table S3. Information pertaining to the location of the primers was obtained from Qiagen (http://www.qiagen.com/geneglobe). β-actin was used as the reference locus for qPCR. Normal human pooled genomic DNA was used as reference DNA (Promega, Madison, USA). All qPCR experiments were performed using the Rotor-Gene 6000™ (Qiagen, Hilden, Germany). Each assay was performed in triplicate in 10 µL reactions containing 5 µL QuantiFast SYBR Green PCR Master Mix (2×; Qiagen); 2 ul QuantiTect Primer assay (10×; Qiagen); and 10 ng of genomic DNA. PCR product amplification was performed according to the following conditions: 1 cycle at 95°C for 10 minutes, 40 cycles, of 95°C for 10 seconds and 60°C for 30 seconds.

To evaluate the mRNA expression levels of candidate genes, we used qRT-PCR. mRNA levels of the candidate gene were compared to those of housekeeper genes. The geometric mean of the relative gene expression of *BAT1*, *SEPT2* and *18s* were used as the comparator reference for RT-PCR. Relative gene expression was calculated using the Pfaffl method [28]. qRT-PCR was carried out using QuantiTect primer sets and 30 ng of cDNA as template. All assays were performed in triplicate in 10 µL reactions containing 5 µL QuantiFast SYBR Green PCR Master Mix (2×; Qiagen) and 2 ul QuantiTect Primer assay (10×; Qiagen).

### Methylation Analysis

#### Microarray

Methylation microarray data (manuscript in preparation) was available for 49 tumor samples from the training set. Genomic DNA was bisulphite converted using an EZ DNA Methylation Kit (Zymo Research, CA, USA) and hybridized to Illumina Infinium Methylation 27 V1.0 microarrays. The β-value was determined as the ratio of methylated fluorescence signal to the combined signal of the methylated and unmethylated alleles, giving a value between 0 and 1.

#### MassARRAY ® EpiTYPER

We employed the MassARRAY® EpiTYPER analysis (Sequenom), for the detection and quantitation of DNA methylation of the promoter regions of *SOCS6*. The EpiTYPER Sequenom Mass Array service was provided by Sequenom, Inc. (Brisbane, Australia) and for each sample, 1.5 µg of DNA in a volume of 30 µL was sent to the service. EpiTYPER is a validated approach in providing a highly quantitative view of CpG dinucleotide methylation, with up to single nucleotide resolution, using the technique of MALDI TOF (Matrix-assisted laser desorption/ionization Time of Flight) mass spectrometry. Primers for *SOCS6* were designed using the Epidesigner software (primers available on request). The *SOCS6* promoter was divided into one or more amplicons, within a region comprising 2500 bp upstream of the transcription start site and the regions analyzed corresponded to annotated CpG islands identified using the UCSC Genome Browser (http://genome.ucsc.edu). Genomic DNA was bisulfite treated using EZ-96 DNA methylation kits (Zymo Research, CA, USA), followed by PCR amplification using primers directed to the promoter regions of *SOCS6*. Amplicons were then subjected to the EpiTYPER assays, the products analyzed by mass spectrometry and methylation ratios obtained using EpiTYPER v1.0.5 software (SEQUENOM). The relative amount of methylation (% methylation) was determined by comparing the signal intensities between the mass signals of methylated and non-methylated template.

### Statistical Analysis

All statistical analyses were performed using SPSS (Version 17, SPSS Inc., Chicago, USA). Fisher's exact test was applied to assess the relationship between copy number as a bivariate categorical variable (above or below the log base 2 threshold) and clinico-pathological characteristics. The Mann-Whitney *U* test was used to measure differences in candidate gene copy number between recurrence and non-recurrence samples. The censored five-year overall survival after surgical resection was estimated using the Kaplan-Meier method and survival differences were analyzed using the log-rank test.

## Results

### aCGH Profile of Lung SCC Tumors and Recurrence Phenotype

In the training and test set subjects, there were no significant differences in clinical or pathological characteristics between those with disease recurrence and those without (Table 1).

**Table 1:** Subject Characteristics.

	Training set			Test set		
	Recurrence SCC, n (%)	Non-Recurrence SCC, n (%)	*p*-value*	Recurrence SCC, n(%)	Non-Recurrence SCC, n(%)	*p*-value*
**Subjects, n**	28	34		20	52	
**Age, years**						
**Median (range)**	67 (39–81)	69 (44–84)		69 (39–82)	69 (44–91)	
**Gender, n(%)**						
Males	21 (75)	25 (73)	1.0	14 (70)	43 (83)	0.33
Females	7 (25)	9 (27)		6 (30)	9 (17)	
**Smoking history, n(%)**						
Never	3 (11)	1 (3)	0.32	2 (10)	4 (7)	0.67
Ever smokers	25 (89)	33 (97)		18 (90)	48 (93)	
Pack years, median (range)	35 (0–100)	38 (0–135)		39 (0–75)	35 (0–243)	
**Stage, n(%)**						
I–II	23 (82)	30 (88)	0.72	14 (70)	44 (85)	0.19
III–IV	5 (18)	4 (12)		6 (30)	8 (15)	
**Tumor differentiation, n(%)**						
Well to moderate	14 (50)	18 (53)	1.0	9 (45)	26 (50)	0.80
Poor	14 (50)	16 (47)		11 (55)	26 (50)	
**Tumor invasion, n(%)**						
Lymphatic	8 (29)	4 (12)	0.12	2 (10)	5 (10)	1.00
Vascular	11 (39)	8 (24)	0.27	10 (50)	15 (29)	0.10
Perineural	3 (11)	0 (0)	0.09	2 (10)	4 (8)	0.67
Pleural	6 (21)	8 (24)	1.00	5 (33)	16 (31)	0.78

*p-value determined using Fisher's exact test.

**Figure 1:**
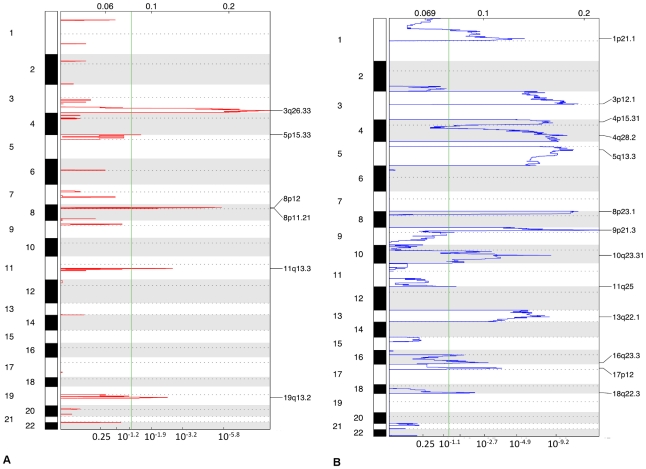
Genomic copy number alterations in lung squamous cell carcinomas. Plots of high-level amplifications (a) and deletions (b) in 62 lung SCCs from GISTIC analysis of aCGH data. X-axis shows the *G* score (top) and false discovery rate (*q* value; bottom) with a green line demarcating a false discovery rate of 0.05. Labels on the right denote the peaks of the most significantly altered regions.

We initially assayed copy number alterations in all 62 tumors of the training set. GISTIC identified nineteen genomic regions (thirteen deletions and six amplifications) with a false discovery rate <0.05 (Figure 1) (Table 2). The frequency of copy number alteration at each of these regions was compared between disease recurrence groups. Only 18q22.3 was significantly different, showing more frequent loss in recurrence compared with non-recurrence tumors (54% vs. 24%, *p* = 0.038) (Table 3). When the tumors were stratified by recurrence phenotype and analyzed separately, GISTIC identified loss of 18q22.3 only in tumors, which recurred, while loss in 1p, 3p, 4q, 5q, 8p, 9p, 10q, 13q, 16q, 17p were common to both phenotypes. Conversely, amplifications in 5p15.33, 8q24.21, 9p21.1 and 19q13.2 were unique to non-recurrence tumors while amplifications in 3q and 8p occurred in both phenotypes (Table S1).

**Table 2:** GISTIC Identified Chromosomal Regions with Copy Number Alterations in Training Set SCC Tumors.

Cytoband	Broad or Focal	Region Limits	Frequency of deletions in test set samples, n (%)	FDR values	GISTIC genes within region	Cancer-associated genes
**Regions with Copy Number Losses**						
9p21.3	Both	21733411–21957547	33 (53)	2.30×10^−18^	**4**	*CDKN2A, CDKN2B*
8p23.1	Broad	3313385–12711819	40 (65)	1.04×10^−13^	**67**	*ANGPT2*
3p12.1	Broad	84414538–85858393	38 (61)	1.04×10^−13^	**6**	*POU1F1*
5q13.3	Broad	76066702–76539988	38 (61)	7.97×10^−13^	**76**	*PIK3R1, HTR1A*
4q28.2	Broad	130278523–134426567	37 (60)	1.06×10^−11^	**12**	*PLK4*
10q23.31	Broad	89540134–89615643	25 (40)	2.67×10^−09^	**4**	*PTEN*
13q22.1	Broad	72396304–72490843	25 (40)	4.32×10^−09^	**7**	
1p21.1	Broad	101664084–102994090	30 (48)	2.41×10^−06^	**8**	*COL11A1*
17p12	Broad	12394069–12459756	24 (39)	1.50×10^−04^	**17**	
4p15.31	Broad	20391110–20848036	35 (56)	4.09×10^−03^	**59**	*BST1, SLIT2, PI4K2B*
16q23.3	Broad	80626349–81449546	23 (37)	1.03×10^−03^	**16**	
18q22.3	Broad	64017241–75522477	24 (39)	4.96×10^−03^	**18**	*SOCS6, CD226, RTTN, MBP*
11q25	Broad	132926079–134452384	17 (27)	2.83×10^−02^	**10**	*ACAD8*
**Regions with Copy Number Gains**						
3q26.33	Focal	183473858–183941579	15 (24)	1.38×10^−13^	**9**	*SOX2, TP73L*
8p12	Focal	38703882–38796309	8 (13)	9.01×10^−09^	**2**	*FGFR1*
19q13.2	Focal	43840068–44336812	4 (6)	2.73×10^−04^	**66**	*AKT2, ECH1*
11q13.3	Focal	68821296–70110392	5 (8)	3.06×10^−02^	**13**	*CCND1, PPFIA1*
5p15.33	Focal	1–1510601	6 (10)	1.46×10^−02^	**21**	*TERT*
8p11.21	Focal	41969702–42416269	2 (3)	4.19×10^−02^	**630**	*TACC1*

**Table 3:** Frequency of Copy Number Alterations of GISTIC Identified Chromosomal Regions in Recurrence and Non-recurrence SCC Training Set Samples.

Cytoband	Loss in SCC Recurrence, n (%)	Loss in SCC Non-Recurrence, n (%)	*p*-value*
**Regions with Copy Number Losses**			
18q22.3	15 (54)	8 (24)	0.038
4q28.2	14 (50)	23 (68)	0.198
1p21.1	11 (39)	19 (56)	0.213
10q23.31	9 (32)	16 (47)	0.301
11q25	6 (21)	11 (32)	0.400
8p23.1	20 (71)	20 (59)	0.425
3p12.1	19 (68)	19 (56)	0.434
5q13.3	17 (61)	21 (62)	0.604
13q22.1	10 (36)	15 (44)	0.606
9p21.3	16 (57)	17 (50)	0.617
17p12	10 (36)	14 (41)	0.795
4p15.31	15 (54)	20 (59)	0.798
16q23.3	10 (36)	13 (38)	1.000
**Regions with Copy Number Gains**			
3q26.33	4 (14)	11 (32)	0.139
19q13.2	1 (4)	3 (9)	0.620
5p15.33	2 (7)	4 (12)	0.681
8p12	4 (14)	4 (12)	1.000
11q13.3	2 (7)	3(9)	1.000
8p11.21	1 (4)	1 (3)	1.000

*p-value determined using Fisher's exact test.

### Candidate Genes within Recurrence-Associated Copy Number Loss at 18q22.3

**Figure 2:**
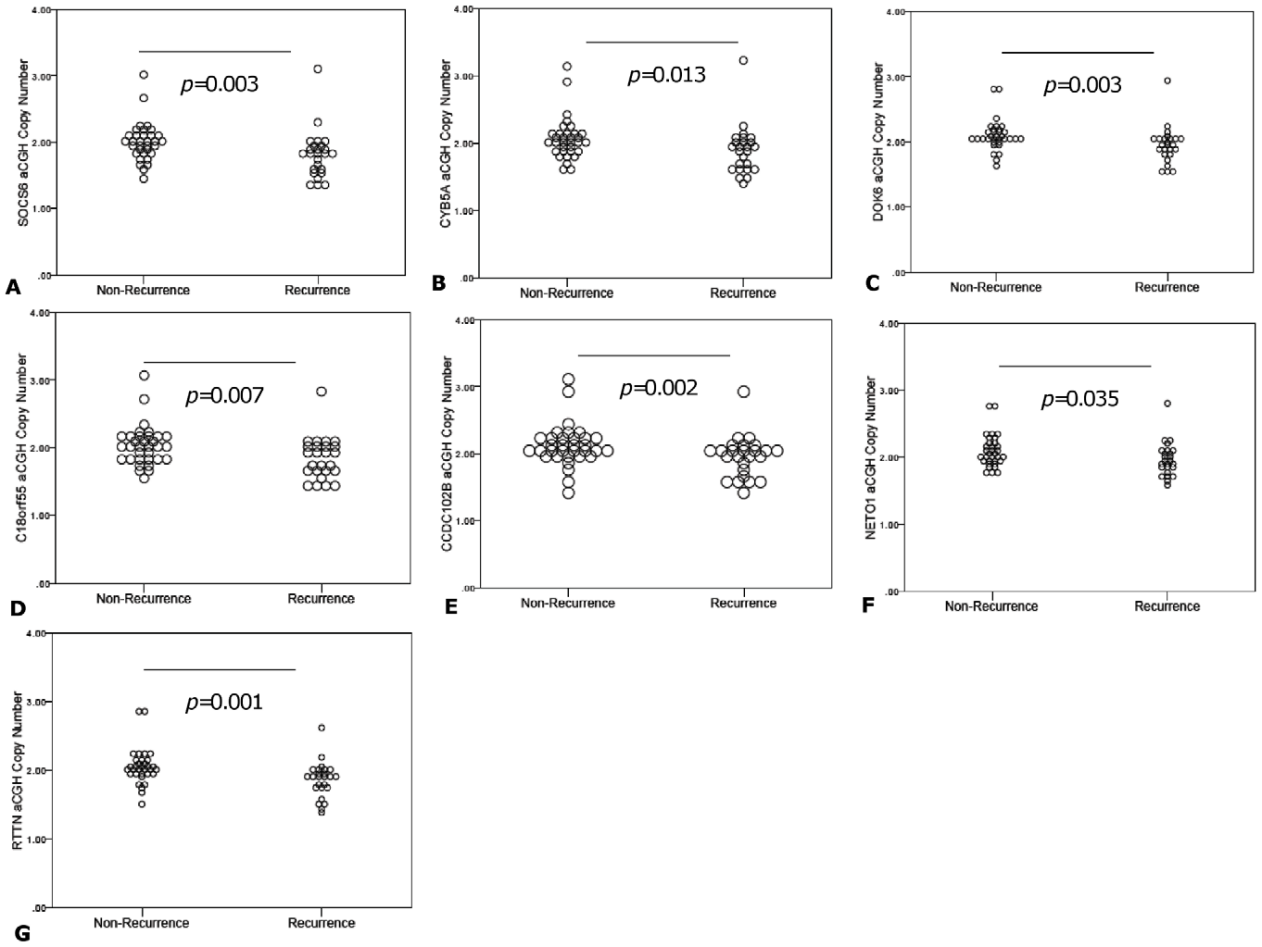
aCGH copy number of genes within 18q22.3 demonstrating preferential loss in SCC recurrence. The Y-axis represents the derived DNA copy number from aCGH log2 normalized data and the X-axis represents the recurrence phenotype. Mann-Whitney *U* test to was used to assess for any differences in copy number between recurrence phenotypes and *p* values<0.05 were deemed significant.

The GISTIC algorithm identified a region of loss within 18q22.3 from 64017241 to 75522477 (Table 2). Within 18q22.3 were 18 candidate genes (Table S2), which were further studied to determine whether they were recurrence-specific. Seven of these (*CYB5A*, *SOCS6*, *DOK6*, *C18orf55*, *CCDC102B*, *NETO1* and *RTTN*) had lower copy number in primary tumors of patients that recurred (Figure 2). Table S2 lists the oligonucleotide probe ID and location for all probes within the 18q22.3 region of interest.

**Figure 3:**
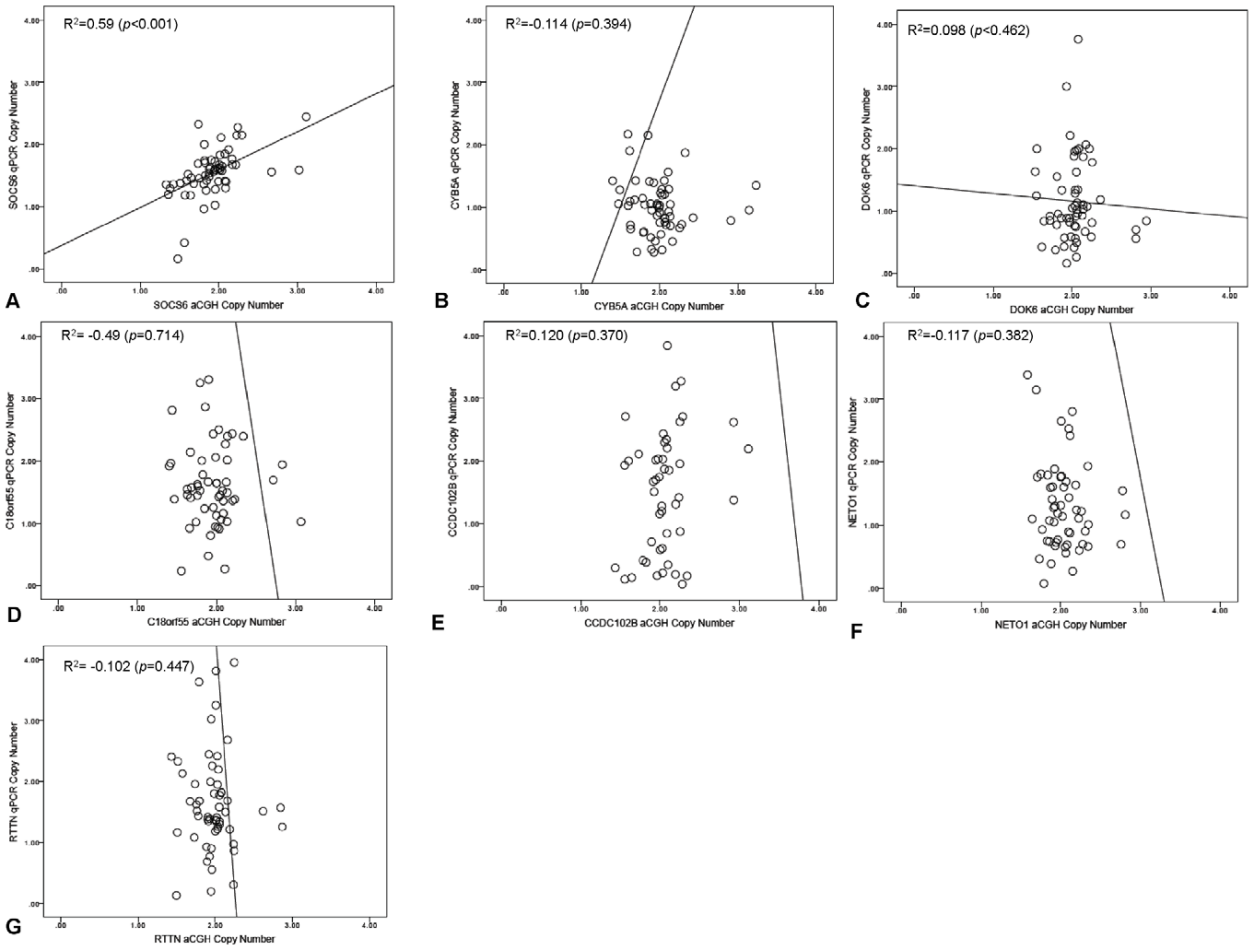
Quantitative PCR (qPCR) validation of array CGH identified candidate genes preferentially lost in SCC recurrence. The Y-axis represents the derived DNA copy number from qPCR normalized to house-keeper genes, *b-actin* while the X-axis represents the DNA copy number derived from aCGH. Pearson's correlation coefficient was used to assess for any relationship between the copy number derived from the methods and *p* values<0.05 were deemed significant. The aCGH copy number of only*SOCS6* (a) was validated by qPCR.

**Figure 4:**
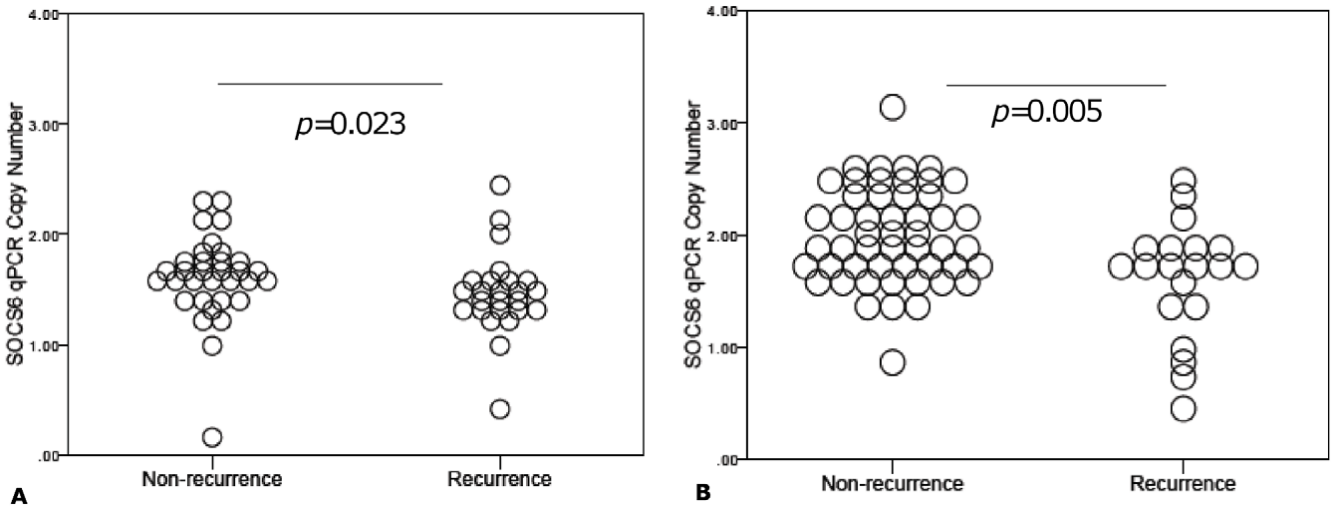
Dot plot of qPCR-derived *SOCS6* copy number (y-axis) is compared to the recurrence phenotype (x-axis) in the training (n = 62) and test set (n = 72) subjects. Figure 4A and 4B represent training set and test set subjects respectively. Mann-Whitney *U* test to was used to assess for any differences in copy number between recurrence phenotypes and *p* values<0.05 were deemed significant.

aCGH based copy number assessment of the seven candidate genes was technically validated by qPCR (Figure 3). A high degree of concordance between aCGH and qPCR data was observed for *SOCS6* (R^2^ = 0.59, *p*<0.001), but *CYB5A*, *DOK6*, *C18orf55*, *CCDC102B*, *NETO1* and *RTTN* showed Pearson coefficients of <0.5 (Figure 3). Given these results, we also tested the association between qPCR-determined copy number in the training and test set tumors. qPCR confirmed significantly lower *SOCS6* copy number in the group with tumor recurrence in both training (*p* = 0.023) (Figure 4A) and test (*p* = 0.005) (Figure 4B) sets.

To confirm that the *SOCS6* copy number alterations were somatically acquired, *SOCS6* copy number was determined by qPCR in the paired normal lung of the training set tumors. The median copy number of *SOCS6* in normal lung was higher than in tumor samples (*p*<0.001) (Figure S1A), and the number of *SOCS6* copies in normal lung did not differ between patients with recurrence and non-recurrence tumors (*p* = 0.321) (Figure S1B).

### SOCS6 mRNA Expression in SCC Tumors

**Figure 5:**
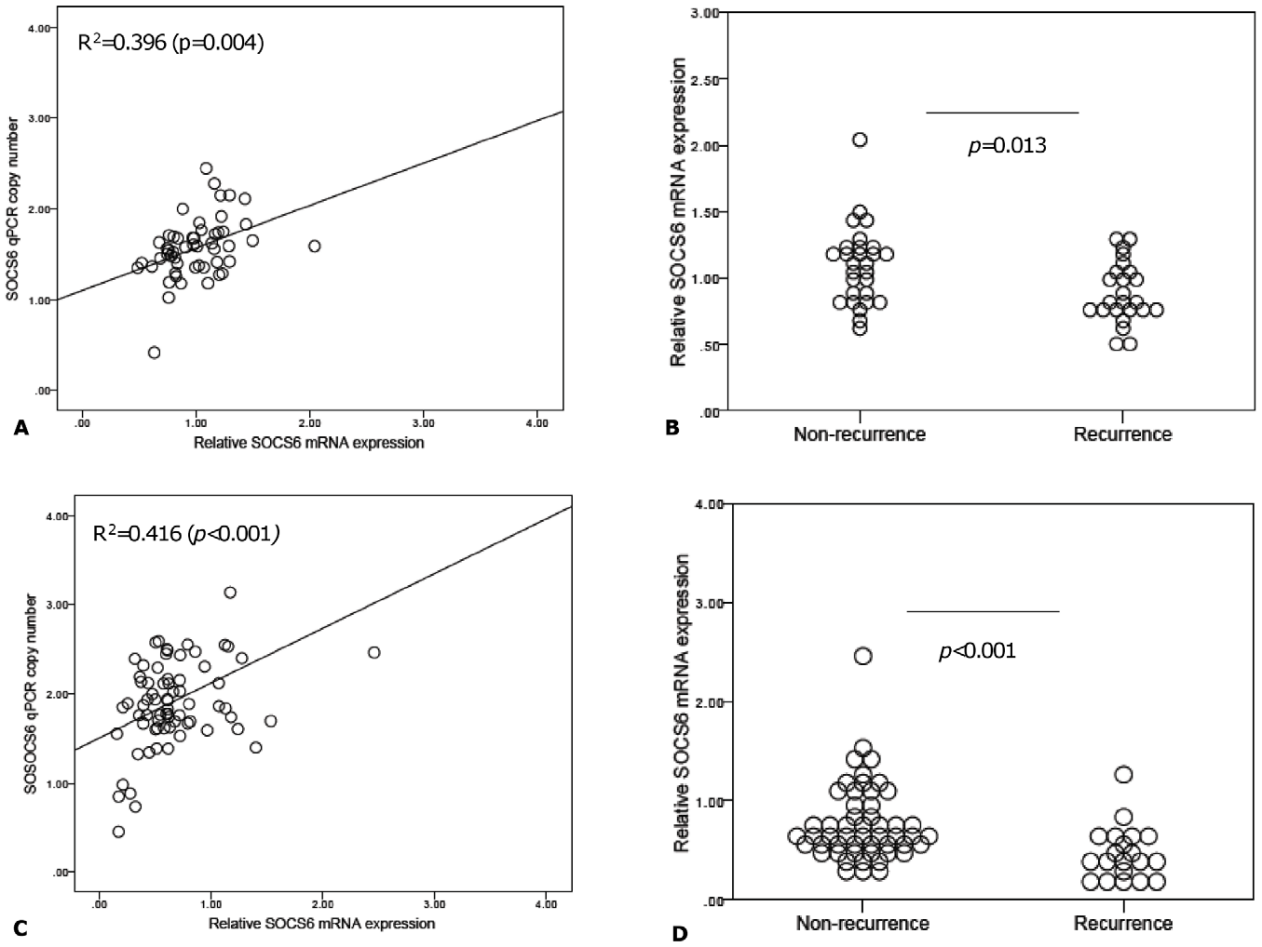
Relationship between *SOCS6* mRNA expression, copy number and recurrence phenotype. Figure 5A and 5C are scatter plots of qRT-PCR derived *SOCS6* mRNA expression (x-axis) and qPCR-derived *SOCS6* copy number (y-axis) in training set (n = 62) (a) and test set (n = 72) (c) respectively. Figure 5B and 5D represent *SOCS6* mRNA levels (y-axis) ad recurrence phenotype (x-axis) in the training and test sets respectively. Pearson's correlation coefficient was used to determine any association between *SOCS6* qPCR derived copy number and mRNA levels. Mann-Whitney *U* test to was used to assess for any differences in copy number between recurrence phenotypes and *p* values<0.05 were deemed significant.

To determine if *SOCS6* DNA copy number was an important regulator of mRNA expression, qRT-PCR was performed in the training and test set tumor cDNA samples. There was a modest correlation between *SOCS6* copy number and mRNA expression in the training set (r^2^ = 0.396, *p* = 0.004) (Figure 5A) and test set (r^2^ = 0.416, *p*<0.001) (Figure 5C). *SOCS6* mRNA expression was significantly lower in recurrence samples in the training (*p* = 0.013, Figure 5B) and test sets (*p* = <0.001, Figure 5D).

### SOCS6 Methylation Status in SCC tumors

An alternative mechanism responsible for reduced mRNA expression, in addition to gene copy number loss, is promoter hypermethylation. We therefore evaluated the methylation status of *SOCS6* in the training set of SCC tumor samples using two independent techniques: Illumina Infinium Methylation microarrays and MassARRAY® EpiTYPER analysis. Methylation microarray analysis of 30 non-recurrence and 19 recurrence tumors from the training set found the methylation index (β) was lower in tumors with *SOCS6* loss (i.e. <2 copies/cell) (n = 29) than in tumors with normal copy number (i.e. ≥2 copies/cell) (n = 20), but the difference was not statistically significant (0.047±0.047 vs. 0.063±0.041, *p* = 0.211). There was no correlation between *SOCS6* methylation and mRNA expression either in samples with <2 *SOCS6* copies/cell (r^2^ = −0.065, *p* = 0.748) or in samples with ≥2 *SOCS6* copies/cell (r^2^ = 0.061, *p* = 0.810).

Quantitation of the degree of *SOCS6* methylation using mass spectrometry of amplification products with the Sequenom EpiTYPER assay was performed in 62 SCC tumor samples from the training set. Five primer sets were designed covering 95 CpG dinucleotides within the promoter region upstream from the transcription site. Thirty-two amplicons failed to provide any analyzable data and another eight had unreliable results and were excluded. The average methylation for the 62 samples across all 55 CpG sites was 9%. There was no difference in the levels of methylation between recurrence and non-recurrence samples (8.9% vs. 9%, *p* = 0.966) and there was no difference between methylation levels according to recurrence phenotype compared to reference DNA (recurrence 8.9% vs. 12%, *p* = 0.189; non-recurrence 9% vs. 12%, *p* = 0.137). As observed in the methylation microarray data, there was a trend for negative correlation between *SOCS6* methylation and mRNA expression in the samples with <2 *SOCS6* copies/cell (R^2^ = −0.377, *p* = 0.092) while this was not the case in samples with ≥2 *SOCS6* copies/cell (R^2^ = 0.232, *p* = 0.210).

### SOCS6 Loss and Decreased mRNA Expression was Associated with Poor Survival in SCC tumors

Training and test subjects were deemed to have ‘*SOCS6* loss’ if their tumors had less than 1.45 copies/cell, as measured by qPCR. The qPCR threshold was derived from the mean copy number minus two standard deviations of the training set tumors with ≥2 copies/cell (aCGH-derived) (n = 26) (*SOCS6* mean copy number ± standard deviation = 2.03±0.29). This approach has been previously used to determine qPCR thresholds for aCGH gene copy number alterations [29].

**Figure 6:**
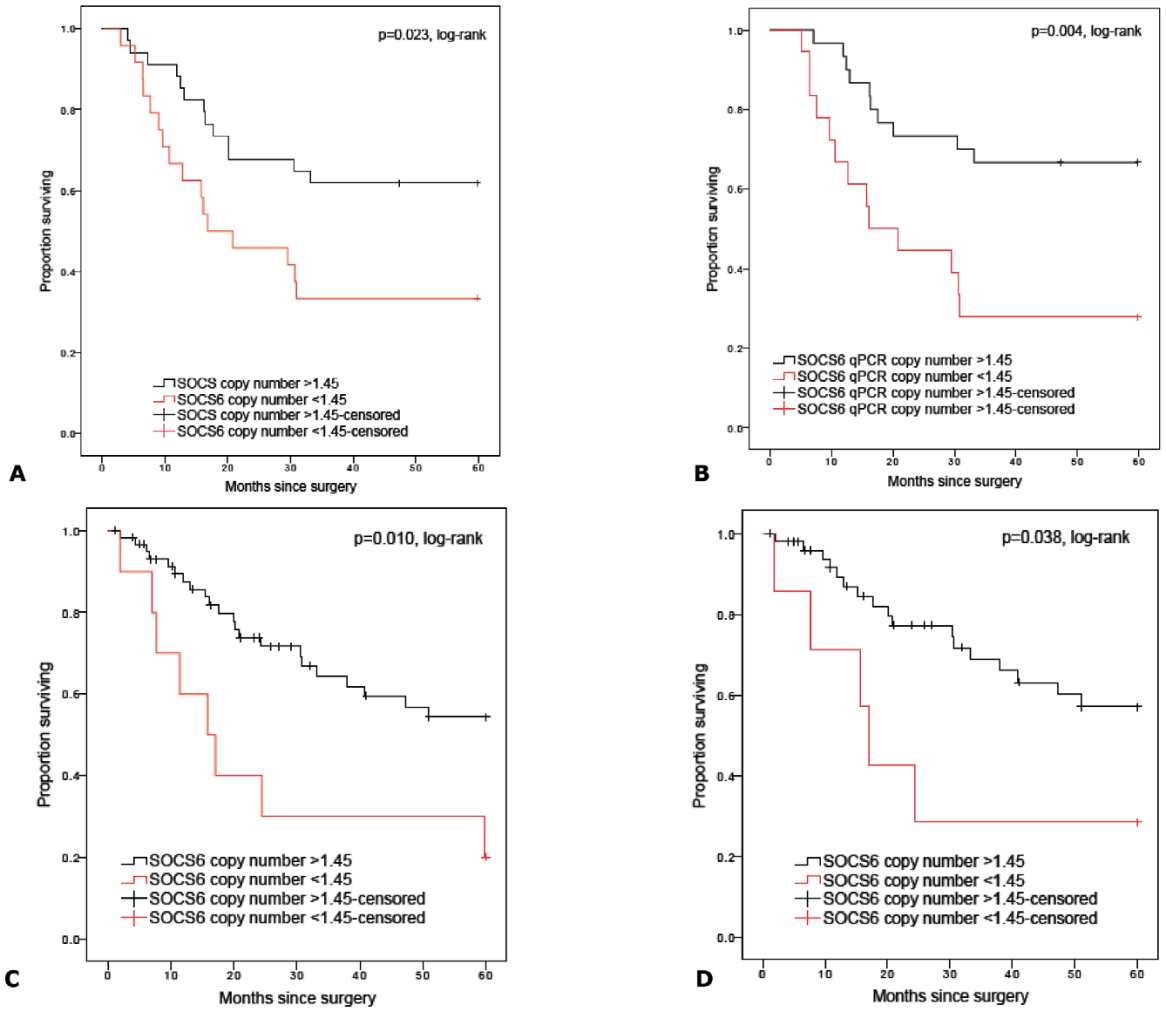
Kaplan-Meier curves of overall survival and *SOCS6* qPCR-derived copy number in study subjects with follow-up duration of 5 years after surgical resection in the training set (n = 62) and test set (n = 72). Figure 6A and 6B represent overall survival in all training set and TNM early stage subjects while 6C and 6D represent all test set and TNM early stage test set subjects. Censored values (+) indicate the last known follow-up time for those subjects still alive after surgical resection.

Evaluation of overall survival at five years post surgery in the training and test set subjects demonstrated that those with ‘*SOCS6* loss’ had significantly worse survival compared to subjects without ‘*SOCS6* loss’ (training set: 30 months vs. 43 months, Log-rank *p* = 0.023; test set: 27 months vs. 43 months, *p* = 0.010) (Figure 6A and 6C, respectively). Stratification by TNM stage found ‘*SOCS6* loss’ was associated with worse survival in early stage (stage I–II) tumors (training set: 28 months vs. 46 months, *p* = 0.004; test set: 27 months vs. 45 months, *p* = 0.038) (Figure 6B and 6D, respectively) but not in the smaller cohort with advanced stage disease (stage III–IV) (training set: 35 months vs. 22 months, *p* = 0.518 and test set: 26 months vs. 34 months, *p* = 0.293, Figure S2A and S2B respectively). ‘*SOCS6* loss’ was also associated with shorter recurrence free survival in both the training (28 months vs. 46 months, Log-rank *p* = 0.007) and test set subjects (28 months vs. 46 months, Log-rank *p* = 0.019) (Figure S3A and S3B, respectively).

Stratification of samples into low *SOCS6* mRNA expression (≤0.85 relative mRNA expression) compared to normal *SOCS6* mRNA expression (>0.85 relative mRNA expression), was associated with worse overall survival (training set: 25 months vs. 44 months, *p* = 0.002; test set: 31 months vs. 48 months, *p* = 0.004) (Figure S4A and S4C, respectively). In the subjects with early stage tumors, decreased *SOCS6* mRNA expression was associated with worse overall survival (training set: 23 months vs. 46 months, *p*<0.001; test set: 35 months vs. 48 months, *p* = 0.033) (Figure S4B and S4D, respectively). However, this was not demonstrable in the small cohort with advanced stage tumors (training set: 28 months vs. 29 months, *p* = 0.973; test set: 12 months vs. 37 months, *p* = 0.101, Figure S2C and S2D respectively).

## Discussion

Recent improvements in clinical outcome in NSCLC has been achieved by using biologically targeted therapies, underpinning the importance of recognizing the molecular heterogeneity of lung cancer [30]. The tumor-node-metastasis (TNM) staging system based on tumor size, nodal involvement and the presence of distant metastases is the current standard for predicting prognosis in NSCLC patients [31], [32]. However, TNM stage cannot fully encompass the heterogeneous biology of NSCLC tumors. In primary lung SCC tumors with well-annotated recurrent disease follow-up, we have tried to identify novel gene/s that may be involved in the pathophysiology of tumor recurrence after surgical resection. In this study, we found that loss of 18q22.3 occurred more often in primary tumors that recurred than those that did not. Located within the 18q22.3 region is *SOCS6*, whose copy number was significantly lower in recurrence tumors compared to the non-recurrence tumors. We also found that DNA copy number loss and that promoter methylation may not regulate *SOCS6* mRNA expression. Importantly, loss of *SOCS6* copy number and reduced mRNA expression had prognostic significance.

The loss of 18q11-23 is a well-recognized marker of poor prognosis in many solid organ malignancies including esophageal squamous cell cancer [33], head and neck squamous cell cancer [34] and colorectal cancer [35]. Recent evidence in gastric cancer suggests that *SOCS6* maybe the candidate gene for the 18q22 copy number alteration and the loss of *SOCS6* appears to be a critical genetic alteration in the development of certain subtypes of gastric cancer [36]. The suppressor of cytokine signaling (SOCS) family comprises eight members SOCS (1–7) and CISH. The SOCS family of proteins negatively regulate the cytokine-induced Janus family tyrosine kinase/signal transducers and activators of transcription signaling pathway, thereby inhibiting the cellular growth and proliferation of tumor cells [37]. Unlike other SOCS family members, *SOCS6* does not interact with JAK2 but has a direct effect on the insulin receptor (IR) and KIT signaling pathways [38], [39]. The deregulation of both insulin and KIT-signaling are known to play an important role in the proliferation of several malignancies [40], [41]. Consequently, tumor cells with loss of *SOCS6* may have increased activation of insulin and KIT-signaling resulting in uncontrolled growth. In gastric cancer, *SOCS6* loss in conjunction with promoter hypermethylation results in transcriptional silencing [36], [42]. Here we show that *SOCS6* mRNA expression is positively correlated with DNA copy number. In our study we found a trend for increased methylation with reduced mRNA expression in the samples with *SOCS6* copy number loss. Further study is warranted in order to better understand the regulatory mechanisms involved in *SOCS6* transcription regulation in lung SCC.

We have shown that the loss of *SOCS6* copy number and corresponding decreases in mRNA expression are related to significantly shorter overall survival, particularly in subjects with early stage SCC tumors. This is clinically important since a prognostic marker for early stage SCCs is definitely needed for the improvement of patients' outcome. Such a prognostic marker may allow clinicians to select the most efficacious adjuvant therapy with consequent improvements in survival. Therefore, if our findings are confirmed in a prospective study, *SOCS6* copy number and/or mRNA expression can be used as a molecular marker for prediction of prognosis in patients with early stage lung SCC. Additionally we have demonstrated that reliable screening for *SOCS6* copy number loss can be performed using the rapid and simple method of qPCR. The small cohort size of subjects with advanced stage SCC, limits our ability to make definitive conclusions about the role of *SOCS6* copy number and mRNA expression as a prognostic biomarker in this cohort of subjects.

Copy number analysis can be useful to identify ‘driver’ (causative of disease) oncogenes or tumor suppressor genes [43]. Causal focal regions of gain (harboring oncogenes) and loss (harboring tumor suppressor genes) in lung SCCs are now being elucidated through high-resolution copy number analyses [14], [18], [23]. These include amplifications in 1p34.2, 2p15, 3q11.2, 3q26.33, 3q29, 4q13.1, 5p15.1, 7p11.2, 8p12, 8q24.21, 9p13.3, 11q13.2, 12p12.1, 14q21.1, 19q12, 19q13.2, 20p12.3 and 22q12.2 and deletions in 3p12.3, 3q24.2, 3q12.1, 4p15.31, 4q32.1, 5p14.2, 5q31.1, 7p11.2, 8p21.3, 9p21.3, 14q21, 17p11.2 and 18q22.1 [14], [18], [23]. In our analysis on a platform of >40,000 elements we found gain in 3q26.33 and loss in 9p21.3 as the most significant alterations in lung SCC, demonstrating the consistency of results from independent studies using high-resolution aCGH platforms. Copy number analysis has also been used to identify genomic alterations associated with metastatic behavior of primary lung SCC [18], [24]. Yan *et al.* used CGH to analyze 21 non-metastatic and 18 metastatic lung SCC tumors and found that when taking advanced stage into consideration, gains on 2p, 20p and losses on 2q, 4q and 18q were associated with metastases [24]. The aCGH study of Boelens *et al*, demonstrated that gains of 7q36, 8p12 and 10q22 were specific to SCC tumors with lymph node metastasis, while gain of 8q22-q24 and loss of 8p23 and 13q21 were specific to SCC tumors that developed distant metastasis within three years of surgical resection [18]. While Boelens *et al.* used aCGH, they did not find an association of 18q22.3 loss with tumor recurrence as we report here, which may reflect differences in the study population, sample size (34 versus 62), and aCGH platforms (6000-element bacterial artificial chromosome-based array versus 44,000-element oligonucleotide array).

Data generated from analysis of high-throughput methods such as aCGH needs to be validated by an alternative method such as qPCR [44]. Inadvertent false discovery due to the large number of probes on micoroarray platforms is the major reason for the need to have biological validation and technical replication. In our study, we noted differences in the aCGH-based and qPCR copy number data. A similar lack of correlation has been noted by others [10]. There are a number of potential reasons for this ranging from false discovery to technical limitations, such as differences between the microarray platform and the qPCR-based copy number assays. For some genes it may relate to limitations of the microarray platform. The Agilent 44B aCGH platform is a platform with 60-mer oligonucleotide probes with resolution of 35–75 kb including coding and noncoding sequences. Low representation of aCGH probes in some regions could prevent accurate quantification of specific genes. Another possible explanation of the poor correlation is the possibility of small SOCS6 intra-genic copy number variations. Microdeletions within a gene may be missed due to the resolution of this Agilent 44B aCGH platform which has an average resolution of approximately 35–75 kb, but will be less likely by newer higher resolution, platforms, such as the 1 M Agilent array [45], [46]. Other studies have used qPCR assays similar to those used in our study to validate aCGH findings [47]. These qPCR assays are designed to span the coding sequence of the candidate gene, while the aCGH probes span both coding and non-coding sequences. Despite this, aCGH and SNP platforms with lower resolution have been able to provide novel insights into disease biology in solid organ malignancies, such as ovarian cancer (50K SNP arrays) [10] and male breast cancer (44B Agilent aCGH arrays) [48].

The tissue samples in this study were macrodissected not microdissected. Microdissection enriches for tumor cells and increases the ability to detect tumor-specific copy number changes. The admixture of normal cells, infiltrating blood and lymphoid cells in our samples may have influenced detection of copy number alterations despite the selection of tumor samples with at least 50% tumor cell content. Despite this limitation, our data showed that loss of *SOCS6* copy number is associated with poor prognosis. This suggests that even despite the presence of non-tumor cells, the detection of *SOCS6* copy number alteration may have potential as a prognostic biomarker. In this study, when we applied the GISTIC algorithm to SCC recurrent and non-recurrent tumors separately, we identified several genomic alterations unique to non-recurrent tumors (5p15.33, 8q24.21, 9p21.1 and 19q13.2). While amplifications in these regions are typically associated with worse clinical outcome in other solid organ malignancies [49], [50], further study is warranted to understand the biological significance, if any, of genomic alterations unique to non-recurrent tumors in lung SCC.

In our study we used an array approach (Illumina BeadsArray Technology) to screen for promoter methylation of *SOCS6*. We then used a quantitative approach (MassARRAY® EpiTYPER analysis) to validate the methylation microarray findings. There are now several approaches available for the study of DNA methylation, some suited for studying single-locus methylation [51] and others for genome-wide approaches [52]. The Illumina BeadArray technology is appropriate technology for genome-wide methylation analysis and the results have been validated against methylation-sensitive PCR, a single gene locus methylation detection method, with high analytical sensitivity [53]. On the other hand, EpiTYPER is a highly accurate quantitative method for that has been validated [54] and used for the evaluation of methylation status in several malignancies including non-small cell lung cancer [55]. Both methods demonstrated that the *SOCS6* gene promoter is not hypermethylated in our samples. However, in the tumors with low copy number of *SOCS6*, there was a trend for increasing methylation, albeit non-significant. This raises the possibility that low copy number and promoter methylation of *SOCS6* may be responsible for reduced mRNA expression. However this will require confirmation in a larger study cohort.

In conclusion, we showed that *SOCS6* located in the genomic locus 18q22.3, has reduced gene copy number and reduced mRNA expression in primary lung SCCs that recur early after surgical resection. Since SOCS family proteins are known to inhibit a potentially important growth-signaling pathway, a tumor suppressor function in lung SCC is a possibility requiring further study to elucidate mechanisms underlying disease recurrence.

## Supporting Information

Figure S1 **qPCR-derived *SOCS6* copy number in normal lung of training set (n = 62).** Figure S1A compares the qPCR-derived *SOCS6* copy number (y-axis) and paired normal lung and tumor tissue (x-axis). Figure S1B compares qPCR-derived *SOCS6* copy number (y-axis) and in training set normal lung recurrence phenotype of the tumor (x-axis) (non-recurrence = 34 and recurrence = 28). Mann-Whitney *U* test to was used to assess for any differences in copy number and *p* values<0.05 were deemed significant.
(TIF)

Figure S2 **Kaplan-Meier curves of overall survival in advanced stage training set (n = 9) and test set (n = 14) study subjects with follow-up duration of 5 years after surgical resection.** Figure S4A and S4B represent qPCR-derived *SOCS6* copy number while Figure S4C and S4D represent SOCS6 mRNA expression and overall survival in TNM advanced stage training set and test set subjects. Censored values (+) indicate the last known follow-up time for those subjects still alive after surgical resection.
(TIF)

Figure S3 **Kaplan-Meier curves of qPCR-derived** ***SOCS6*** **copy number and recurrence-free survival in study subjects with follow-up duration of 5 years after surgical resection.** Figure S2A represents training set subjects (n = 62) and Figure S2B represents test set subjects (n = 72). Censored values (+) indicate the last known follow-up time for those subjects still alive after surgical resection.
(TIF)

Figure S4 **Kaplan-Meier curves of overall survival and relative** ***SOCS6*** **mRNA expression in training set (n = 62) and test set (n = 72) study subjects with follow-up duration of 5 years after surgical resection.** Figure S3A and S3B represent overall survival in all training set and TNM early stage subjects while Figure S3C and S3D represent all test set and TNM early stage test set subjects. Censored values (+) indicate the last known follow-up time for those subjects still alive after surgical resection.
(TIF)

Table S1 **GISTIC Identified Chromosomal Regions with Copy Number Alterations in Non-recurrence and Recurrence SCC Tumors.**
(DOC)

Table S2 **RefSeq Genes Contained Within the GISTIC Identified Cytoband 18q22.3.**
(DOC)

Table S3 **aCGH Probes and QuantiTect (Qiagen) Primers used for qPCR.**
(DOC)
