# Supplementary figures and images for: Array-Comparative Genomic Hybridization Reveals Loss of SOCS6 Is Associated with Poor Prognosis in Primary Lung Squamous Cell Carcinoma
Source: PLoS One. 2012 Feb 17;7(2):e30398. doi: 10.1371/journal.pone.0030398 (PMC3281847; doi:10.1371/journal.pone.0030398)

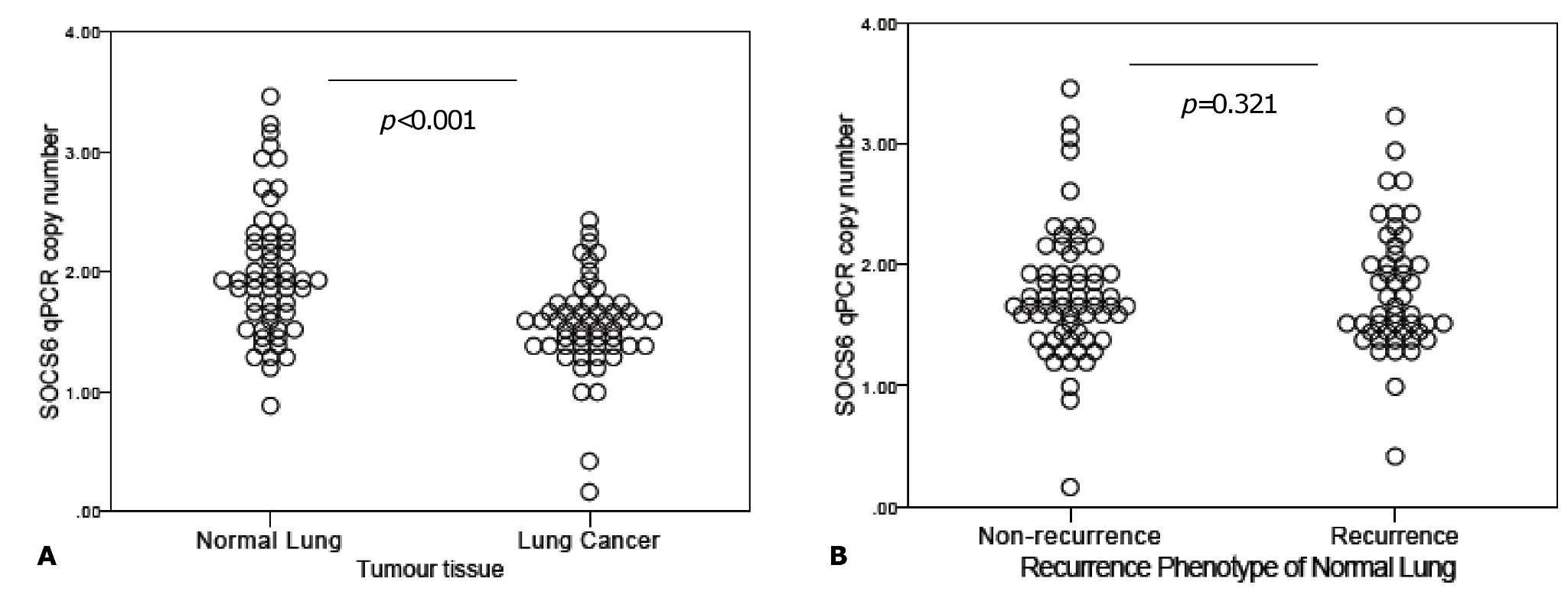

Supplement: Figure S1 — qPCR-derived SOCS6 copy number in normal lung of training set (n = 62). Figure S1A compares the qPCR-derived SOCS6 copy number (y-axis) and paired normal lung and tumor tissue (x-axis). Figure S1B compares qPCR-derived SOCS6 copy number (y-axis) and in training set normal lung recurrence phenotype of the tumor (x-axis) (non-recurrence = 34 and recurrence = 28). Mann-Whitney U test to was used to assess for any differences in copy number and p values<0.05 were deemed significant. (TIF) [file pone.0030398.s001.tif]

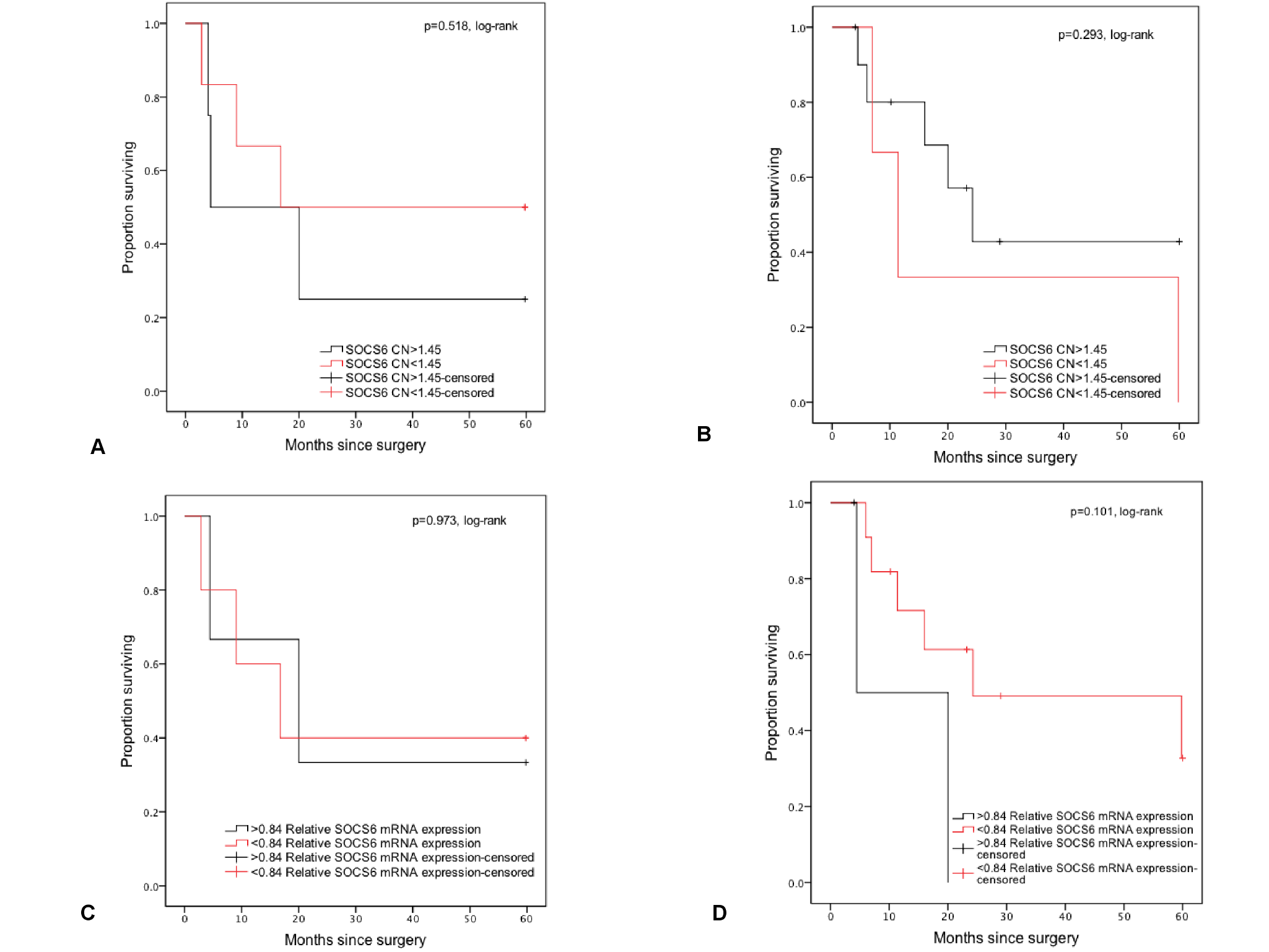

Supplement: Figure S2 — Kaplan-Meier curves of overall survival in advanced stage training set (n = 9) and test set (n = 14) study subjects with follow-up duration of 5 years after surgical resection. Figure S4A and S4B represent qPCR-derived SOCS6 copy number while Figure S4C and S4D represent SOCS6 mRNA expression and overall survival in TNM advanced stage training set and test set subjects. Censored values (+) indicate the last known follow-up time for those subjects still alive after surgical resection. (TIF) [file pone.0030398.s002.tif]

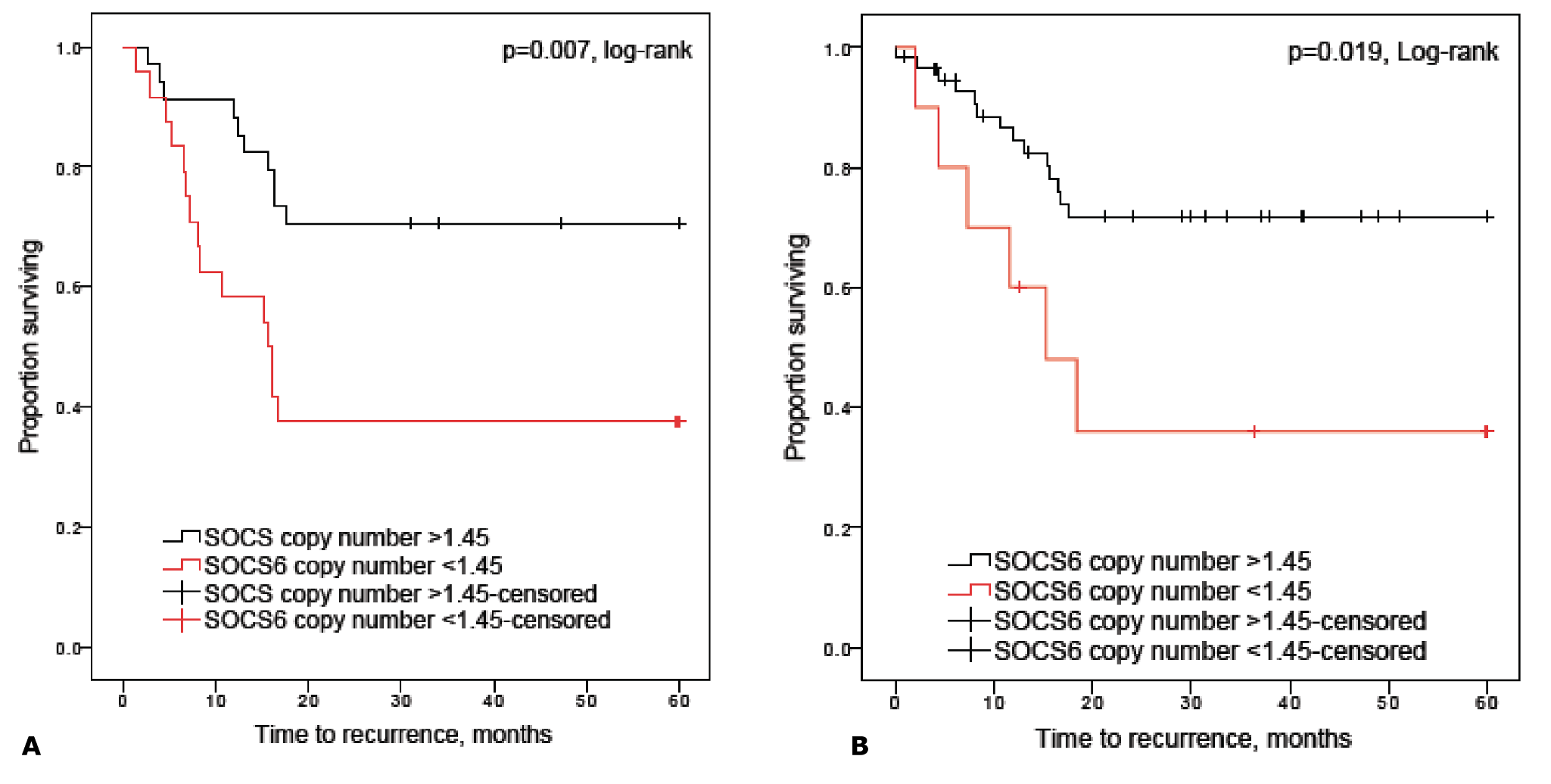

Supplement: Figure S3 — Kaplan-Meier curves of qPCR-derived SOCS6 copy number and recurrence-free survival in study subjects with follow-up duration of 5 years after surgical resection. Figure S2A represents training set subjects (n = 62) and Figure S2B represents test set subjects (n = 72). Censored values (+) indicate the last known follow-up time for those subjects still alive after surgical resection. (TIF) [file pone.0030398.s003.tif]

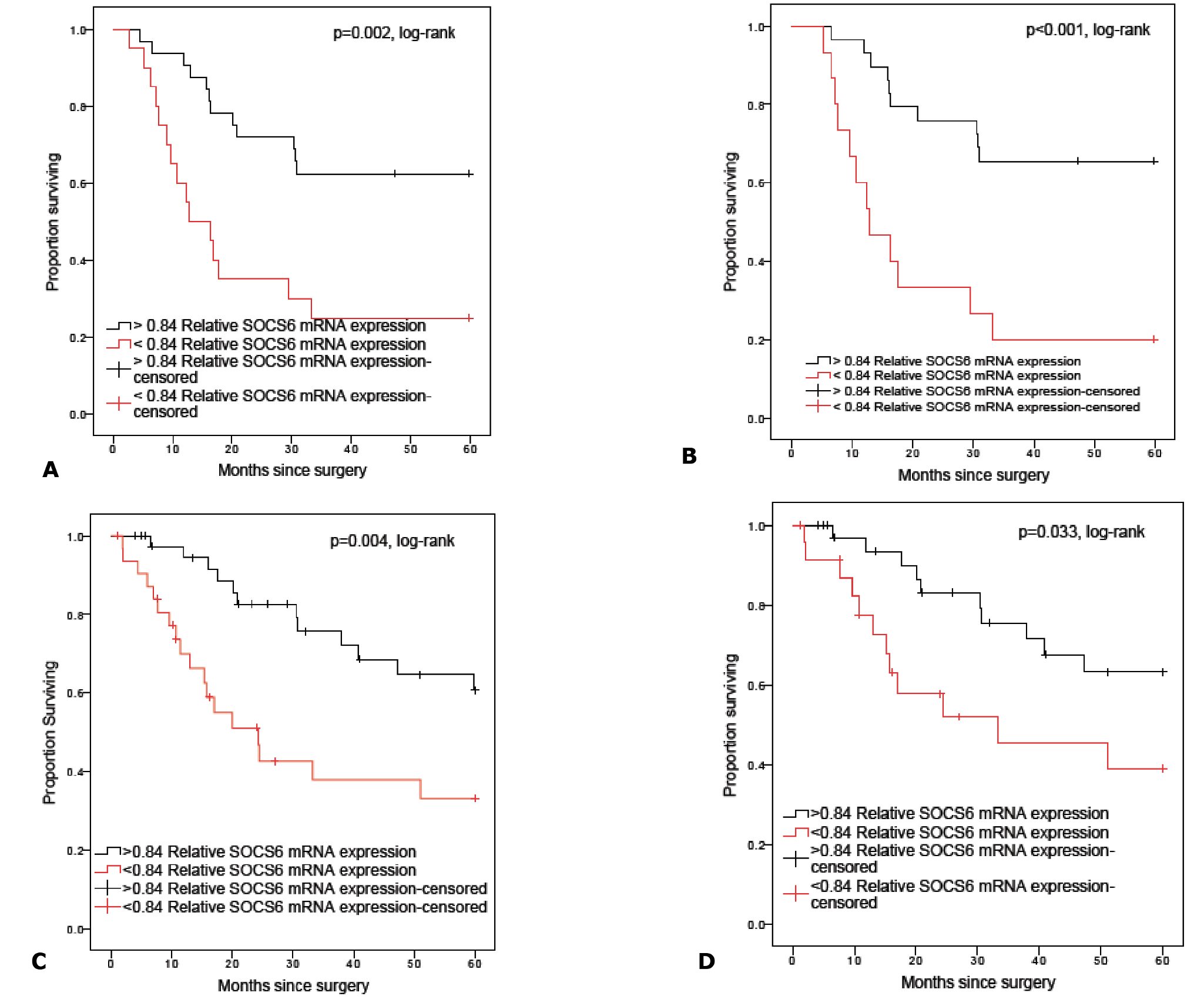

Supplement: Figure S4 — Kaplan-Meier curves of overall survival and relative SOCS6 mRNA expression in training set (n = 62) and test set (n = 72) study subjects with follow-up duration of 5 years after surgical resection. Figure S3A and S3B represent overall survival in all training set and TNM early stage subjects while Figure S3C and S3D represent all test set and TNM early stage test set subjects. Censored values (+) indicate the last known follow-up time for those subjects still alive after surgical resection. (TIF) [file pone.0030398.s004.tif]
