# Supplementary material for: Array-Comparative Genomic Hybridization Reveals Loss of SOCS6 Is Associated with Poor Prognosis in Primary Lung Squamous Cell Carcinoma
Source: PLoS One. 2012 Feb 17;7(2):e30398. doi: 10.1371/journal.pone.0030398 (PMC3281847; doi:10.1371/journal.pone.0030398)
Supplement: Table S1 — GISTIC Identified Chromosomal Regions with Copy Number Alterations in Non-recurrence and Recurrence SCC Tumors. (DOC) [file pone.0030398.s005.doc]

| **Table S1: GISTIC Identified Chromosomal Regions with Copy Number Alterations in Non-recurrence and recurrence SCC Tumors** | | | | | | |
| --- | --- | --- | --- | --- | --- | --- |
| **Copy Number Alteration** | **Recurrence** |  |  | **Non-Recurrence** | | |
|  | **Cytoband** | **Broad or focal** | **FDR value** | **Cytoband** | **Broad or focal** | **FDR value** |
| **Deletions** | 1p13.3 | Broad | 1.18E-02 | 1p21.1 | Broad | 2.92E-04 |
|  | 3p12.1 | Both | 1.11E-08 | 3p14.1 | Broad | 9.46E-07 |
|  | 4q15.2 | Broad | 1.56E-03 | 4q28.2 | Broad | 1.18E-07 |
|  | 5q13.3 | Broad | 4.35E-06 | 5q12.3 | Broad | 9.46E-07 |
|  | 8p23.3 | Broad | 6.35E-07 | 8p23.1 | Broad | 9.46E-07 |
|  | 9p21.3 | Both | 6.53E-13 | 9p21.3 | Broad | 9.46E-07 |
|  | 10q23.2 | Broad | 6.02E-03 | 10q23.31 | Broad | 3.83E-06 |
|  | 13q14.3 | Broad | 1.56E-03 | 13q22.1 | Broad | 2.77E-06 |
|  | 16q23.3 | Broad | 2.95E-02 | 16q22.3 | Broad | 2.57E-02 |
|  | 17p12 | Broad | 3.83E-02 | 17p12 | Broad | 1.24E-02 |
|  | 18q22.3 | Broad | 1.41E-03 |  |  |  |
|  |  |  |  |  |  |  |
| **Amplifications** | 3q28 | Focal | 1.03E-02 | 3q26.33 | Focal | 3.77E-12 |
|  | 8p11.23 | Focal | 1.03E-02 | 5p15.33 | Focal | 2.45E-02 |
|  |  |  |  | 8p12 | Focal | 1.94E-06 |
|  |  |  |  | 8q24.21 | Focal | 1.33E-02 |
|  |  |  |  | 9p21.1 | Focal | 4.34E-02 |
|  |  |  |  | 19q13.2 | Focal | 4.09E-04 |
|  |  |  |  |  |  |  |
