# Supplementary material for: Array-Comparative Genomic Hybridization Reveals Loss of SOCS6 Is Associated with Poor Prognosis in Primary Lung Squamous Cell Carcinoma
Source: PLoS One. 2012 Feb 17;7(2):e30398. doi: 10.1371/journal.pone.0030398 (PMC3281847; doi:10.1371/journal.pone.0030398)
Supplement: Table S2 — RefSeq Genes Contained Within the GISTIC Identified Cytoband 18q22.3. (DOC) [file pone.0030398.s006.doc]

| **Table S2: RefSeq Genes Contained Within the GISTIC Identified Cytoband 18q22.3** | | | | |
| --- | --- | --- | --- | --- |
| **Probe ID** | **Genbank** | **Gene symbol** | **Gene description** | **Literature evidence supporting role in tumorigenesis** |
| A_14_P107107, A_14_P108151 | NM_004232 | *SOCS6* | Suppressor of cytokine signaling 6 | Data support the importance of loss-of-function of *SOCS6* as a frequent event in gastric tumorigenesis  Study demonstrated that hypermethylation of the *SOCS6* promoter is one of the mechanisms for the epigenetic regulation of SOCS6 expression . |
| A_14_P136515, A_14_P119564, A_14_P138647, A_14_P202562 | NM_173630 | *RTTN* | rotatin | Loss of *Dnam-1* gene copy number might be indicators of worse prognosis in patients with colorectal cancer . |
| A_14_P121789 | NM_001480 | *GALR1* | galanin receptor 1 | *GALR1* is a tumor suppressor gene in head and neck squamous cell carcinoma. Deregulation of *GALRI* may lead to unregulated proliferation and neoplastic transformation |
| A_14_P138397, A_14_P201016 | NM_002385 | *MBP* | myelin basic protein | There was a statistically significant inverse correlation between myelin basic protein (*MBP*) expression and proliferation index in pilocyticastrocytomas; expression of MBP was also related to progression-free survival . |
| A_14_P131675, A_14_P121642, A_14_P133915 | NM_148923 | *CYB5A* | cytochrome b5 type A (microsomal) | Nil |
| A_14_P201157, A_14_P118306, A_14_P117925 | NM_007345 | *ZNF236* | zinc finger protein 236 | Nil |
| A_14_P116444, A_14_P126600, A_14_P130872, A_14_P117392, A_14_P134400 | NM_152721 | *DOK6* | docking protein 6 | Nil |
| A_14_P118749 | NM_006566 | *CD226* | CD226 molecule | Nil |
| A_14_P130772 | NM_005786 | *SDCCAG33 (TSHZ1)* | teashirt zinc finger homeobox 1 | Nil |
| A_14_P200509  A_14_P103860 | NM_014177 | *C18orf55* | chromosome 18 open reading frame 55 | Nil |
| A_14_P101122, A_14_P123148, A_14_P133279, A_14_P101638, A_14_P202418, A_14_P108618, A_14_P134173 | NM_017757 | *ZNF407* | zinc finger protein 407 | Nil |
| A_14_P133122, A_14_P101737, A_14_P114298 | NM_018235 | *CNDP2* | CNDP dipeptidase 2 (metallopeptidase M20 family) | Nil |
| A_14_P101720 | NM_032649 | *CNDP1* | carnosinedipeptidase 1 (metallopeptidase M20 family) | Nil |
| A_14_P104639, A_14_P129345 | NM_175907 | *ZADH2* | zinc binding alcohol dehydrogenase domain containing 2 | Nil |
| A_14_P102390, A_14_P125669, A_14_P127888 | NM_024781 | *C18orf14 (CCDC102B)* | coiled-coil domain containing 102B | Nil |
| A_14_P201953, A_14_P132805 | NM_138966 | *NETO1* | neuropilin (NRP) and tolloid (TLL)-like 1 | Nil |
| A_14_P138114 | NM_182511 | *CBLN2* | cerebellin 2 precursor | Nil |
| A_14_P123288 | NM_152676 | *FBXO15* | F-box protein 15 | Nil |

# References

1. Lai R-H, Hsiao Y-W, Wang M-J, Lin H-Y, Wu C-W, Chi C-W, et al. SOCS6, down-regulated in gastric cancer, inhibits cell proliferation and colony formation. Cancer Letters. 2010;288(1):75-85.

2. Storojeva I, Boulay J-L, Ballabeni P, Buess M, Terracciano L, Laffer U, et al. Prognostic and Predictive Relevance of DNAM-1, SOCS6 and CADH-7 Genes on Chromosome 18q in Colorectal Cancer. Oncology. 2004;68:246-55.

3. Kanazawa T, Kommareddi PK, Iwashita T, Kumar B, Misawa K, Misawa Y, et al. Galanin Receptor Subtype 2 Suppresses Cell Proliferation and Induces Apoptosis in p53 Mutant Head and Neck Cancer Cells. Clinical Cancer Research. 2009 April 1, 2009;15(7):2222-30.

4. Takei H, Yogeswaren S, Wong K-K, Mehta V, Chintagumpala M, Dauser R, et al. Expression of oligodendroglial differentiation markers in pilocytic astrocytomas identifies two clinical subsets and shows a significant correlation with proliferation index and progression free survival. Journal of Neuro-Oncology. 2008;86(2):183-90.
