# Supplementary material for: Array-Comparative Genomic Hybridization Reveals Loss of SOCS6 Is Associated with Poor Prognosis in Primary Lung Squamous Cell Carcinoma
Source: PLoS One. 2012 Feb 17;7(2):e30398. doi: 10.1371/journal.pone.0030398 (PMC3281847; doi:10.1371/journal.pone.0030398)
Supplement: Table S3 — aCGH Probes and QuantiTect (Qiagen) Primers used for qPCR. (DOC) [file pone.0030398.s007.doc]

| **Table S3: aCGH Probes and QuantiTect (Qiagen) Primers used for qPCR** | | | | | |
| --- | --- | --- | --- | --- | --- |
| **Gene** | **Gene Position** | **aCGH Probe** | | **QuantiTect Primer** | |
|  |  | **Probe ID** | **Position** | **Probe ID** | **Probe location*** |
| *SOCS6* | 66,107,117-66,148,414 | A_14_P107107,  A_14_P108151 | 66108961-66109020  66134551-66134610 | QT00202244 | 66,143,870 |
| *CYB5A* | 70,071,508-70,110,201 | A_14_P131675,  A_14_P121642,  A_14_P133915 | 70079500-70079559  70097895-70097954  70103203-70103262 | QT00066479 | 70,107,892 |
| *DOK6* | 65219264-65667303 | A_14_P116444,  A_14_P126600,  A_14_P130872,  A_14_P117392,  A_14_P134400 | 65342290-65342349  65417600-65417600  65516619-65516619  65596369-65596369  65659843-65659843 | QT02307823 | 65660144 |
| *C18orf55* | 69966726-69977177 | A_14_P200509  A_14_P103860 | 69969346-69969399  69976665-69976724 | QT00086555 | 66,976,833 |
| *CCDC102B* | 64552949-64873406 | A_14_P102390,  A_14_P125669,  A_14_P127888 | 64656931-64656990  64749099-64749158  64829240-64829299 | QT00041167 | 64,685,389 |
| *NETO1* | 68560651-68686164 | A_14_P201953,  A_14_P132805 | 68574272-68574331  68683878-68683937 | QT00069510 | 68,634,818 |
| *RTTN* | 65822025-66023942 | A_14_P136515,  A_14_P119564,  A_14_P138647,  A_14_P202562 | 65837332-65837391  65893622-65893681  65985040-65985099  66006378-66006437 | QT00008141 | 65,862,259 |

* Probe locations calculated based on information provided by Qiagen (www.qiagen.com).
